# Linezolid in the treatment of severe intraabdominal infection: A STROBE-compliant retrospective study

**DOI:** 10.1097/MD.0000000000030038

**Published:** 2022-08-19

**Authors:** Deyuan You, Yuexiang Su, Xuri Sun, Jianbao Wang, Yuxin Zheng, Yuqi Liu

**Affiliations:** a Department of Respiratory and Critical Care Medicine, Fujian Respiratory Medical Center, The Second Affiliated Hospital of Fujian Medical University, Quanzhou City, Fujian Province, China; b Department of Critical Care Medicine, Quanzhou Anxi County Hospital, Quanzhou City, Fujian Province, China.

**Keywords:** linezolid, procalcitonin, sepsis, severe intra-abdominal infection, thrombocytopenia

## Abstract

Safety concerns over bone marrow suppression and thrombocytopenia may inhibit the use of linezolid to treat intraabdominal infection (IAI). To evaluate the effectiveness, safety, and prognosis of linezolid in the treatment of severe IAI (SIAI). Patients were divided into a linezolid group and nonlinezolid group according to whether linezolid was prescribed. Subgroup analysis (thrombocytopenia treated with linezolid group (I), and thrombocytopenia treated with nonlinezolid group (II) also was performed. We evaluated the effectiveness of linezolid by analyzing the changes in white blood cells (WBC) and procalcitonin, evaluated safety by analyzing the changes in platelet counts, and evaluated patient outcomes by analyzing the length of hospital stay, the length of ICU stay, and the rates of clinical improvement. Sixty-six adult SIAI patients were treated with anti-gram-positive (G^+^) bacteria drugs for more than 7 days from January 1, 2014, to December 31, 2020. The length of hospital stay, the length of ICU stay, and the rates of clinical improvement were not significantly different between the linezolid group and nonlinezolid group. On the 15th day after anti-G ^+^ bacteria treatment, the WBC of the linezolid group was significantly lower than in the nonlinezolid group (9.00 ± 4.30 vs 13.1 ± 6.19, *P* < .05). The time for a statistical difference in the decrease of procalcitonin in the linezolid group was earlier than in the nonlinezolid group (day 6 vs day 7, *P* < .05). There was no statistically significant difference in the changes of platelet counts in the subgroup I (*P* > .05), but compared with the baseline data (day 0), the time for the statistical difference in the increase of platelets in thrombocytopenia treated with linezolid group was earlier (day 5 vs day 6, *P* < .05). There was no statistical difference in the changes of platelets in subgroup II (*P >* .05). In the treatment of severe intraabdominal infection in a single-center, retrospective study, linezolid was not inferior to other antibiotics in patient clinical outcomes or seral WBC and procalcitonin values. Linezolid also induced no evident bone marrow suppression or thrombocytopenia. Linezolid is a good choice for treatment of SIAI.

## 1. Introduction

intraabdominal infection (IAI) is the second most common infectious disease among intensive care unit (ICU) patients.^[1]^ Severe intraabdominal infection (SIAI) is often associated with sepsis, septic shock, abdominal compartment syndrome, and multiple organ dysfunction; it is difficult to treat, and it has a high mortality rate (about 20%).^[1]^ Therefore, timely and effective infection source control and antibiotic application are important.^[1]^ SIAI is mostly a mixed infection, in IAI caused by abdominal trauma, in the ascites culture, Gram-negative(G^-^) bacteria, Gram-positive(G^+^)bacteria and fungi accounted for 53.5% (159/297), 44.1%(131/297) and 0.7% (2/297), respectively.^[2]^ A total of 118 strains were isolated from the samples of 65 IAI patients with cirrhosis, with positive test results. Among the 118 strains, 74 of them were G^-^ bacteria (62.71%), 41 were G ^+^ bacteria (34.75%), and 3 were fungus (2.54%).^[3]^ With the most common gram-positive bacteria being *Enterococcus* (16.90%) and methicillin-resistant *Staphylococcus aureus* (MRSA) (3.90%).^[2]^

The drugs currently clinically used to treat *Enterococcus* and MRSA include vancomycin, teicoplanin, teicoplanin and linezolid. And the sensitivity to linezolid is almost 100%. Linezolid has unique pharmacological properties, it has low molecular weight (337.35 Daltons) gives it good tissue penetration so that it can easily penetrate the peritoneum into the peritoneal cavity and achieve an effective therapeutic concentration, and it has less impact on liver and kidney function. Consider the pathogens and drug susceptibility of SIAI, the pharmacological properties and advantages of linezolid, the antibiotic is a good choice for treating SIAI. However, several factors may interfere with use of linezolid for the treatment of SIAI: 1) The product’s guidelines recommend using it only for treatment of infection by MRSA but not as a first-line anti-gram positive bacteria option.^[4]^ 2) The linezolid instructions highlight its bone marrow suppression (such as thrombocytopenia), and a study in Japan pointed out that thrombocytopenia developed in 48.4% of patients during linezolid therapy,^[5]^ the pooled incidences of thrombocytopenia and anemia were 9% (95% confidence interval (CI), 3–18%) and 4% (95% CI, 0–12%),^[6]^ which may make clinicians wary of using the drug for treating SIAI patients who have thrombocytopenia and is a common manifestation of sepsis. To further understand the clinical application of linezolid in SIAI, we conducted this retrospective analysis to evaluate the effectiveness, safety, and prognosis of linezolid in the treatment of SIAI.

## 2. Materials and Methods

### 2.1. Study participants

**Figure 1.**
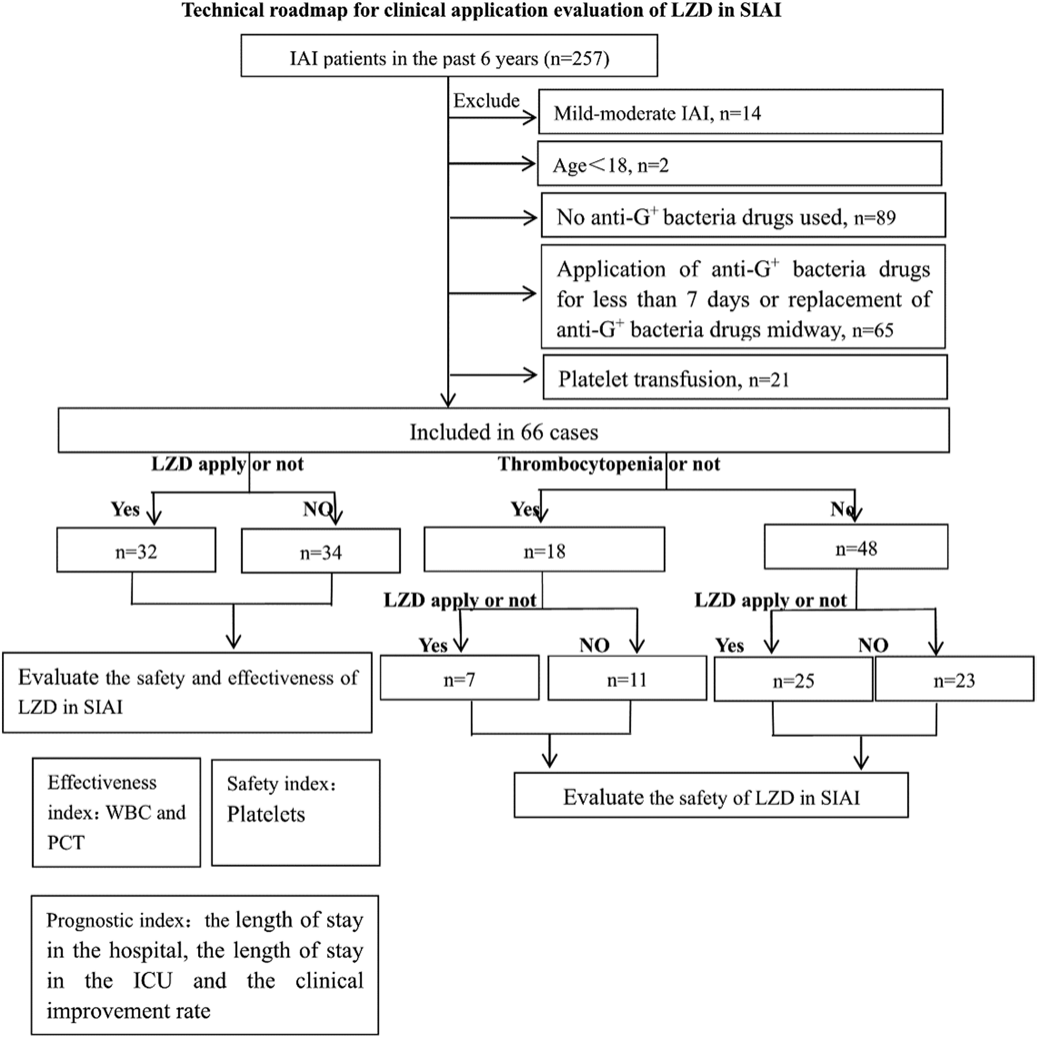
Technical roadmap for clinical application evaluation of LZD in SIAI. Two hundred fifty-seven IAI patients admitted to the ICU of the Second Affiliated Hospital of Fujian Medical University were selected retrospectively from January 1, 2014, to December 31, 2020. Inclusion criteria were (1) SIAI patients with APACHE II score above 10; SIAI combined with sepsis; and SIAI combined with acute gastrointestinal dysfunction grade III-IV. The diagnosis of SIAI could be made if any one of these 3 criteria was reached. (2) Use of anti-Gram positive (anti-G^+^) bacteria drugs for more than 7 days. Exclusion criteria were age < 18 years of age; application of anti-G ^+^ bacteria drugs for <7 days, or replacement of anti-G ^+^ bacteria drugs midway; no anti-G ^+^ bacteria drugs used; platelet transfusion given during treatment. Sixty-six cases met the inclusion and exclusion criteria. Sixty-six adult SIAI patients who were treated with anti-Gram positive (G+) bacteria drugs for more than 7 days from January 1, 2014, to December 31, were divided into a linezolid treatment group and nonlinezolid treatment group. Subgroup analysis (thrombocytopenia treated with linezolid group and thrombocytopenia treated without linezolid group) analysis was also performed. We evaluated the effectiveness of linezolid in SIAI treatment by analyzing the changes in white blood cells (WBC) and procalcitonin, evaluated the safety by analyzing the changes in platelet counts, and evaluated the outcome (prognosis) by analyzing the length of hospital stay, the length of ICU stay, and the clinical improvement rate. Clinical improvement is defined as stable vital signs after treatment, normal inflammatory index, and transfer of the patient out of the ICU.

Two hundred fifty-seven IAI patients admitted to the ICU of the Second Affiliated Hospital of Fujian Medical University were selected retrospectively from January 1, 2014, to December 31, 2020. Inclusion criteria were 1) SIAI patients with APACHE II score above 10; SIAI combined with sepsis; and SIAI combined with acute gastrointestinal dysfunction grade III-IV. The diagnosis of SIAI could be made if any one of these 3 criteria was reached. 2) Use of anti-gram-positive (anti-G^+^) bacteria drugs for more than 7 days becasus a high incidence of linezolid-induced thrombocytopenia was even detected among the patients that had received linezolid therapy for < 7 days.^[5]^ Exclusion criteria were age < 18 years of age; application of anti-G ^+^ bacteria drugs for <7 days, or replacement of anti-G ^+^ bacteria drugs midway; no anti-G ^+^ bacteria drugs used; platelet transfusion given during treatment. Sixty-six cases met the inclusion and exclusion criteria (Fig. 1).

### 2.3. Methods

Sixty-six adult SIAI patients who were treated with anti-Gram positive (G^+^) bacteria drugs for more than 7 days from January 1, 2014, to December 31, were divided into a linezolid treatment group and nonlinezolid treatment group. Subgroup analysis (thrombocytopenia treated with linezolid group and thrombocytopenia treated without linezolid group) analysis was also performed. We evaluated the effectiveness of linezolid in SIAI treatment by analyzing the changes in white blood cells (WBC) and procalcitonin, evaluated the safety by analyzing the changes in platelet counts, and evaluated the outcome (prognosis) by analyzing the length of hospital stay, the length of ICU stay, and the clinical improvement rate. Clinical improvement is defined as stable vital signs after treatment, normal inflammatory index, and transfer of the patient out of the ICU (Fig. 1).

### 2.4. Ethical review

This study was reviewed by the Ethical Review Committee of the Second Affiliated Hospital of Fujian Medical University, number [2021] 289.

### 2.5. Statistical analysis

All statistical analyses were performed with SPSS for Windows version 22.0 (SPSS Japan Inc., Tokyo, Japan). Data are expressed as mean ± standard ($\bar{\text{X}}$ ± s) deviation values, unless otherwise specified. Continuous variables were analyzed with the unpaired Student t-test, while categorical variables were analyzed with the χ^2^ test. P-values of < .05 were considered significant.

## 3. Results

### 3.1. Study population

The gender, age, Acute Physiological and Chronic Health II (APACHE II score), Sequential Organ Failure Assessment Score, Community-acquired intraabdominal infection/Hospital-associated infection (CA-IAI/HA-IAI) score, white blood cells (WBC), procalcitonin, platelet count, and other baseline values of the 2 patient groups (linezolid treated and nonlinezolid treated) are presented in Table 1. All comparisons were *P* > .05, indicting no statistically significant difference between the groups.

**Table 1:** Comparison of general data between linezolid group and nonlinezolid group.

General data	Linezolid group(n = 32)	nonlinezolid group (n = 34)	*P* values
Male (n, %)	23, 71.88	24, 75.00	χ^2^ = 0.01, *P* > .05
CA-IAI/HA-IAI	18/14	25/9	χ^2^ = 2.17, *P* > .05
Age (mean ± SD, yr)	60.00 ± 13.73	56.50 ± 17.50	*P =* .38
APACHE II score (mean ± SD)	16.09 ± 4.20	16.79 ± 5.03	*P =* .55
SOFA score (mean ± SD)	6.19 ± 3.20	7.68 ± 3.43	*P =* .08
WBC (mean ± SD, ×10^9^/L)	14.64 ± 9.57	13.76 ± 5.63	*P =* .66
Procalcitonin (mean ± SD, ng/ml)	30.80 ± 39.44	30.80 ± 37.11	*P* = .77
Platelets (mean ± SD, ×10^9^/L)	211.32 ± 139.00	155.59 ± 109.21	*P* = .09

APACHE II score = acute physiological and chronic health scores II, CA-IAI/HA-IAI = community-acquired intraabdominal infection/Hospital-associated intraabdominal infection, SOFA = sequential organ failure assessment.

In the treatment of SIAI, there are many effective indicators: subjective indicators (pain degree and mental state) and objective indicators (WBC, procalcitonin, th1/th2 cell counts, blood pressure, heart rate). Because of limitations of the retrospective analysis, we used only changes in the WBC and procalcitonin values in the laboratory evaluation of the effectiveness of linezolid. The results in Table 2 reveal that on the 15th day after anti-G ^+^ bacteria treatment, the WBC of the linezolid group was significantly lower than that of the nonlinezolid group (9.00 ± 4.30 vs 13.1 ± 6.19, *P* < .05). Also, the decrease of WBC in the linezolid group at 15 days compared with day 0 was significantly decrease, whereas there was no significant change in the WBC during that interval change in the nonlinezolid group. There was no significant difference in the procalcitonin value between the 2 groups at the same days of treatment. However, compared with day 0, the procalcitonin value in the linezolid group declined slightly but significantly sooner (day 6 vs day 7, *P* < .05).

**Table 2:** Comparison of WBC and procalcitonin between linezolid group and nonlinezolid group ($\bar{\text{X}}$ ± s).

Time	WBC (mean ± SD, ×10^9^/L)		*P*-values	Procalcitonin (mean ± SD, ng/ml)		*P*-values
	linezolid group (n = 32)	Nonlinezolid group (n = 34)		Linezolid group (n = 32)	Nonlinezolid group (n = 34)	
Day 0	14.64 ± 9.57	13.76 ± 5.63	*P* = .66	30.80 ± 39.44	30.80 ± 37.11	*P* = .77
Day 1	13.48 ± 7.10	14.66 ± 5.20	*P* = .46	37.14 ± 42.04	20.62 ± 26.54	*P* = .24
Day 2	12.20 ± 5.47	13.61 ± 2.33	*P* = .31	30.16 ± 30.85	22.96 ± 31.27	*P* = .59
Day 3	11.20 ± 5.15	14.65 ± 6.99	*P* = .21	17.24 ± 25.40	21.57 ± 33.25	*P* = .70
Day 4	12.45 ± 5.01	14.39 ± 7.87	*P* = .29	11.98 ± 21.63	19.75 ± 31.34	*P* = .49
Day 5	12.24 ± 5.82	14.08 ± 8.83	*P* = .35	8.74 ± 18.90	13.45 ± 19.99	*P* = .59
Day 6	12.68 ± 6.57	15.40 ± 14.55	*P* = .36	5.55 ± 9.82*	7.96 ± 13.76	*P* = .62
Day 7	12.71 ± 6.50	13.72 ± 6.87	*P* = .56	3.21 ± 3.82*	6.18 ± 8.96†	*P* = .29
Day 9	11.30 ± 5.25	14.33 ± 7.14	*P* = .07	1.56 ± 1.48*	2.34 ± 3.05†	*P* = .51
Day 11	11.28 ± 5.64	14.07 ± 5.84	*P* = .10	1.25 ± 1.23*	3.05 ± 3.42†	*P* = .12
Day 13	11.62 ± 4.55	13.09 ± 5.54	*P* = .33	0.77 ± 0.97*	1.22 ± 1.45†	*P* = .47
Day 15	9.00 ± 4.30*	13.14 ± 6.19	*P* = .02‡	1.21 ± 0.73*	1.10 ± 1.56†	*P* = .30

WBC = White blood cell.

* Indicates *P* < .05, there was a statistical difference in linezolid Group compared with Day0.

† Indicates *P* < 0.05, there was a statistical difference in nonlinezolid Group compared with Day0.

‡ Indicates *P* < .05, there was a statistical difference between 2 groups.

To further understand the efficacy of linezolid in SIAI treatment, we analyzed patients’ length of hospital stay, length of ICU stay, and clinical improvement rate (Table 3). The 2 patient groups were not significantly different in these criteria.

**Table 3:** Comparison of patient outcomes between linezolid group and nonlinezolid group($\bar{\text{X}}$ ± s).

Term	Linezolid Group(n = 32)	Nonlinezolid group (n = 34)	*P*-values
Length of hospital stay (mean ± SD, days)	37.72 ± 17.38	37.82 ± 23.97	*P* = .98
Length of ICU stay (Mean ± SD, days)	23.69 ± 13.48	31.06 ± 23.09	*P* = .13
Improvement rate	25 (73.53%)	22 (64.71%)	χ^2^ = 0.62, *P >*.05

Besides the therapeutic effect, safety is an important consideration in determining the clinical utility of linezolid in the treatment of SIAI. Indexes such as platelet counts, liver function, kidney function, and nutritional status can reflect the safety of the drug. There was no significant difference in liver function, renal function, nutritional status and coagulation function between the 2 groups. Then, we compared the changes in platelet count between the 2 patient groups. For this purpose, we divided the patients into 2 subgroups: Subgroup I: 18 patients with thrombocytopenia, and Subgroup II: 48 patients with normal platelet counts. In Subgroup I, (thrombocytopenia), 7 patients were treated with linezolid, and 11 were treated with nonlinezolid antibiotics. In Subgroup II, (normal platelet counts), 25 patients were treated with linezolid, and 23 were treated with nonlinezolid antibiotics. The patients in the 2 subgroups were similar (*P* > .05) in gender, age, APACHE II score, and SOFA score (Table 4). At 5 days of treatment, platelet counts in Subgroup I patients treated with linezolid had risen significantly compared with baseline values(*P* < .05), whereas a similar rise in counts occurred at day 6 in patients treated with nonlinezolid antibiotics (Table 5).

**Table 4:** Comparison of general data between 2 subgroups ($\bar{\text{X}}$ ± s).

General data	Subgroup I		*P*-values	Subgroup II		*P*-values
	Thrombocytopenia treated with linezolid group (n = 7)	Thrombocytopenia treated with nonlinezolid group (n = 11)		Normal platelets treated with linezolid group (n = 25)	Normal platelets treated with nonlinezolid group (n = 23)	
Male (n, %)	4, 57.14	7,63.64	*P>*.05	19/6	17/6	*P >*.05
Age (mean ± SD, yr)	64.57 ± 12.09	57.27 ± 15.64	*P* = .34	58.72 ± 13.89	56.13 ± 18.31	*P* = .59
APACHE II score (mean ± SD)	18.43 ± 4.03	15.73 ± 5.26	*P* = .29	15.44 ± 4.01	17.30 ± 4.84	*P* = .16
SOFA score (mean ± SD)	8.29 ± 3.53	8.64 ± 4.42	*P* = .87	5.60 ± 2.83	7.22 ± 2.72	*P* = .05
Improvement rate	5 (71.43%)	5 (45.45%)	*P >*.05	19 (76.00%)	17 (73.91)	*P W>*.05

APACHE II score = acute physiological and chronic health scores II, SOFA = sequential organ failure assessment.

**Table 5:** Comparison of platelet counts between 2 subgroups.

Time	Subgroup I (mean ± SD, ×10^9^/L)		*P*-values	Subgroup II (mean ± SD, ×10^9^/L)		*P*-values
	Thrombocytopenia treated with linezolid group (n = 7)	Thrombocytopenia treated with nonlinezolid group (n = 11)		Normal platelets treated with linezolid group (n = 25)	Normal platelets treated with nonlinezolid group (n = 23)	
Day 0	28.57 ± 29.47	53.36 ± 26.15	*P* = .10	264.63 ± 110.10	209.14 ± 97.33	*P* = .09
Day 1	22.50 ± 16.57	59.36 ± 35.21	*P* = .08	226.09 ± 108.94	205.96 ± 111.91	*P* = .55
Day 2	47.29 ± 26.42	54.55 ± 41.56	*P* = .70	228.25 ± 144.06	219.41 ± 134.06	*P* = .83
Day 3	62.00 ± 45.18	60.64 ± 40.03	*P* = .95	218.16 ± 152.97	215.85 ± 140.44	*P* = .96
Day 4	56.33 ± 28.81	70.30 ± 51.36	*P* = .58	233.25 ± 150.01	233.45 ± 153.02	*P* = .99
Day 5	74.29 ± 29.25*	91.55 ± 61.30	*P* = .52	248.91 ± 162.33	284.90 ± 173.75	*P* = .49
Day 6	72.40 ± 52.29*	120.18 ± 79.36†	*P* = .23	262.67 ± 146.35	290.48 ± 181.27	*P* = .58
Day 7	86.57 ± 70.72*	163.30 ± 93.14†	*P* = .10	290.35 ± 164.31	320.30 ± 186.19	*P* = .57
Day 9	125.29 ± 100.90*	200.67 ± 117.83†	*P* = .22	314.58 ± 193.97	341.26 ± 193.14	*P* = .66
Day 11	201.67 ± 122.43*	184.75 ± 112.21†	*P* = .81	320.57 ± 243.96	371.71 ± 247.59	*P* = .56
Day 13	222.83 ± 128.33*	170.67 ± 89.06†	*P* = .47	321.05 ± 289.71	396.07 ± 239.74	*P* = .43
Day 15	260.00 ± 150.77*	162.33 ± 75.57†	*P* = .24	249.59 ± 165.44	350.47 ± 212.54	*P* = .15

* Indicates *P* < .05, there was a statistical difference in thrombocytopenia treated with linezolid group compared with Day 0.

† Indicates *P* < .05, there was a statistical difference in thrombocytopenia treated with nonlinezolid group compared with Day 0.

It is worth noting that antibiotic-associated diarrhea, such as Clostridium difficile-associated diarrhea(CDAD), may occur after prolonged use of almost all antibiotics, including linezolid. Because the use of antibiotics can alter the normal flora of the gut, resulting in the overgrowth of *C. difficile*. If a patient develops severe diarrhea during or after linezolid treatment, CDAD should be considered and linezolid may need to be discontinued. More than this, it has been reported that even some CDADs do not appear until 2 months after linezolid administration.^[7]^ In our study, patients with SIAI were often complicated with paralytic ileus, intestinal fistula, the frequency of diarrhea was not high, and no growth of *Clostridium difficile* was detected in stool examination.

## 4. Discussion

Based on the data from this study, the main findings of this retrospective review of the treatment of severe intrabdominal infections with linezolid is that the antibiotic was not inferior to other antibiotic regimens in patient outcomes or laboratory values. No significant difference was found in patients’ length of hospital stay, length of ICU stay, and clinical improvement rate between patients treated with linezolid and those treated with other antimicrobial medications. Trends in WBC values were in favor of linezolid, with significantly lower counts after 15 days’ treatment and a trend towards greater decline from pretreatment values to day 15 values in the patients treated with linezolid.

In evaluation of the safety of linezolid, the drug did not cause a decrease in platelet counts, and, in fact, platelet counts in the SIAI patients treated with linezolid increased more than did those treated with other agents (*P* < .05). This is an important finding because the fear of bone marrow suppression and thrombocytopenia has been a drawback to broader use of linezolid in the treatment of infections. Indeed, the linezolid product instructions highlight its bone marrow suppression (such as thrombocytopenia), which may make clinicians wary of using the drug for treating SIAI patients who have thrombocytopenia, a common manifestation of sepsis.

The commonly used antibiotics for the treatment of G ^+^ bacterial infections are vancomycin, teicoplanin, tigecycline, and linezolid,^[8]^ which have unique pharmacological properties. Vancomycin has strong bactericidal ability, and it is effective against almost all multi-drug-resistant G ^+^ bacteria. However, drug-resistant strains, such as vancomycin-resistant *Staphylococcus aureus* (VRSA), and *vancomycin-resistant enterococcus* (VRE) are a serious problem.^[9]^ Also, vancomycin has poor tissue penetration and has otic and renal toxicity. SIAI often has associated renal insufficiency due to septic shock and acute coronary syndrome. Renal function and vancomycin blood concentration must be monitored closely and the dose adjusted promptly.^[10]^ Teicoplanin has the same antibacterial spectrum as vancomycin, but with better effects on VRSA and VRE^[11]^ and less otic and renal toxicity. However, the protein binding capacity of teicoplanin is about 90%–95%. Since SIAI patients often have hypoproteinemia, their conjugated teicoplanin value may be low and the free teicoplanin high. Consequently, drug metabolism is accelerated, resulting in a drug-time curve with the area under the curve significantly reduced and antiinfective effects poor. Tigecycline can widely cover G ^+^ cocci, G^-^ bacilli, and anaerobic bacteria. It was officially approved for the treatment of complex IAI in adults in 2010.^[12]^ However, Phase III and Phase IV clinical studies found that patients using tigecycline had increased all-cause mortality,^[12]^ so the guidelines^[1]^ do not recommend tigecycline as a routine, empiric treatment for SIAI.

Linezolid is a new generation of synthetic oxazolidinone antibacterial drugs. Because of its unique antibacterial mechanism, bacteria cannot easily develop cross-resistance to linezolid and other antimicrobials. Linezolid can be used to treat infections caused by G ^+^ bacteria, including MRSA and VRE.^[13]^ Since linezolid is not yet indicated for the treatment of IAI, there are just a few studies on the pharmacodynamics and pharmacokinetics of linezolid in that condition. However, linezolid has low molecular weight and good tissue penetration, and in animal models, it has penetrated abdominal abscesses with effective drug concentrations.^[14]^ Some^[15]^ have found that after the first dose of linezolid, its concentration in peritoneal dialysate is > 4 µg/ml (minimum inhibitory concentration, MIC), and with treatment duration, the average concentration in the peritoneal dialysate increases, so that after 8 hours its concentration is still higher than the MIC. Although IAI is not an indication in the product’s instructions for the use of linezolid, clinical application of linezolid in China^[16,17]^ has had good effects in IAI, and guidelines of the Journal of Practical Surgery^[1]^ state that linezolid can be used to treat SIAI infected by MRSA. However, the number of SIAI infected by MRSA has been small, and SIAI usually requires early and powerful antiinfective treatment; if one waits for the drug susceptibility results to treat MRSA before choosing linezolid, it may affect the treatment effect and prognosis..

As the largest comprehensive ICU in Quanzhou City, our department admits many SIAI patients with complex disease types and serious illnesses. We have chosen linezolid as an anti-G ^+^ bacteria drug because its treatment effect has been good, and the occurrence of thrombocytopenia has been low. Thus, we adopted this “unpopular” method to explore the application of linezolid in SIAI; the number of patients was modest, and the representativeness was relatively strong.

The mechanism of thrombocytopenia associated with SIAI has not been fully clarified; it may be related to infection destruction, bone marrow suppression, immune imbalance, microthrombosis, and consumption.^[18]^ Whatever the mechanism, the serious infection must be controlled as soon as possible. In view of the pharmacodynamic characteristics of linezolid, we believe that it is a good choice for the treatment of SIAI. Nevertheless, concern about linezolid’s putative effects on the bone marrow and platelets persists, especially in patients with SIAI combined with thrombocytopenia. However, the literature did not clarify whether the thrombocytopenia was related to the SIAI or to linezolid. If linezolid is discontinued without evidence that the drug is responsible for the diminished platelet counts, its effectiveness against the infection may be jeopardized.

This study has limitations. It is a single-center, retrospective, and observational study, with a modest number of cases. The pharmacokinetics of linezolid in SIAI could not be monitored. In the future, we plan to conduct prospective, double-blind clinical trials, even nationwide studies, and clinical efficacy/pharmacokinetic monitoring to further determine the clinical application of linezolid in SIAI.

## 5. Conclusions

Based on our research data, in the treatment of severe intraabdominal infection in a single-center, retrospective study, linezolid was not inferior to other antibiotics. Linezolid did not induce evident bone marrow suppression or thrombocytopenia. Linezolid appears to be an effective and safe antibiotic for the treatment of severe intraabdominal infection. However, rigorously controlled, multi-center, prospective studies are needed to verify the effectiveness and safety of linezolid in the treatment of severe intraabdominal infection.

## Acknowledgments

The authors thank the doctors and nursing staff of the intensive care units for their excellent patient care. We thank LetPub (www.letpub.com) for linguistic assistance and presubmission expert review.

## Author contributions

Study concept and design: Deyuan You, Yuexiang Su, Xuri Sun, Jianbao Wang, Yuxin Zheng, Yuqi Liu.

Acquisition, analysis, or interpretation of data: Deyuan You, Yuexiang Su, Xuri Sun, Jianbao Wang, Yuxin Zheng, Yuqi Liu.

Drafting of the manuscript: Deyuan You.

Critical revision: Yuqi Liu, Deyuan You.

Statistical analysis: Deyuan You, Yuexiang Su, Jianbao Wang.

Study supervision: Deyuan You, Yuexiang Su, Yuqi Liu.

## References

[1] Mazuski JE Tessier JM May AK. The surgical infection society revised guidelines on the management of intra-abdominal infection.. Surg Infect (Larchmt). 2017;18:1–76. 28085573 10.1089/sur.2016.261

[2] Zhang S Ren L Li Y. Bacteriology and drug susceptibility analysis of pus from patients with severe intra-abdominal infection induced by abdominal trauma. Exp Ther Med. 2014;7:1427–31. 24940451 10.3892/etm.2014.1609 PMC3991502

[3] Yan Y Ye Q Liu L. Characteristics of pathogenic bacteria in intra-abdominal infection and risk factors for septic shock in patients with liver cirrhosis.. Am J Transl Res. 2022;14:1742–9. 35422921 PMC8991129

[4] Wu X Ren J. Guidelines for the diagnosis and treatment of abdominal infection in China (2019 edition). Chin J Pract Surg. 2020;40:1–16.

[5] Hanai Y Matsuo K Ogawa M. A retrospective study of the risk factors for linezolid-induced thrombocytopenia and anemia. J Infect Chemother. 2016;22:536–42. 27321773 10.1016/j.jiac.2016.05.003

[6] Kato H Hagihara M Asai N. A systematic review and meta-analysis of myelosuppression in pediatric patients treated with linezolid for Gram-positive bacterial infections.. J Infect Chemother. 2021;27:1143–50. 33727025 10.1016/j.jiac.2021.03.003

[7] Montoya M Detorres O. Antimicrobial selection and its impact on the incidence of Clostridium difficile-associated diarrhea.. J Pharm Pract. 2013;26:483–7. 23940122 10.1177/0897190013499524

[8] Xiong Y-M Rao X. Clinical and microbiological characteristics of patients with complicated intra-abdominal infections in intensive care unit. Curr Med Sci. 2020;40:104–9. 32166671 10.1007/s11596-020-2152-x

[9] Reveles KR Mortensen EM Attridge RT. Comparative-effectiveness of vancomycin and linezolid as part of guideline-recommended empiric therapy for healthcare-associated pneumonia. BMC Res Notes. 2015;8:450. 26382940 10.1186/s13104-015-1396-1 PMC4573683

[10] Ye Z-K Chen Y-L Chen K. Therapeutic drug monitoring of vancomycin: a guideline of the Division of Therapeutic Drug Monitoring, Chinese Pharmacological Society. J Antimicrob Chemother. 2016;71:3020–5. 27494905 10.1093/jac/dkw254

[11] Tascini F Flammini S Leonildi A. Comparison of teicoplanin and vancomycin in vitro activity on clinical isolates of *Staphylococcus aureus*. J Chemother. 2012;24:187–90. 23040680 10.1179/1973947812Y.0000000026

[12] Solomkin JS Mazuski JE Bradley JS. Diagnosis and management of complicated intra-abdominal infection in adults and children: guidelines by the Surgical Infection Society and the Infectious Diseases Society of America. Clin Infect Dis. 2010;50:133–64. 20034345 10.1086/649554

[13] Rybak JM Barber KE Rybak MJ. Current and prospective treatments for multidrug-resistant gram-positive infections. Expert Opin Pharmacother. 2013;14:1919–32. 23876168 10.1517/14656566.2013.820276

[14] Schulin T Thauvin-Eliopoulos C Moellering RC. Activities of the oxazolidinones Linezolid and eperezolid in experimental intra-abdominal abscess due to Enterococcus faecalis or vancomycin-resistant Enterococcus faecium. Antimicrob Agents Chemother. 1999;43:2873–6. 10582874 10.1128/aac.43.12.2873 PMC89579

[15] DePestel DD Peloquin CA Carver PL. Peritoneal dialysis fluid concentrations of Linezolid in the treatment of vancomycin-resistant Enterococcus faecium peritonitis. Pharmacotherapy. 2003;23:1322–6. 14594349 10.1592/phco.23.12.1322.32702

[16] Wang X Wan C. A case report of linezolid in the treatment of refractory biliary tract infection. Chin J Pract Intern Med. 2009;29:581–2.

[17] Zhang Y Wu X. A case report of linezolid in the treatment of intra-abdominal infection. Chin J Pract Intern Med. 2009;29:580–1.

[18] Brotfain E Schwartz A Boniel A. Clinical outcome of critically ill patients with thrombocytopenia and hypophosphatemia in the early stage of sepsis.. Anaesthesiol Intensive Ther. 2016;48:294–9. 27834985 10.5603/AIT.a2016.0053

